# Supplementary material for: Degradation of arouser by endosomal microautophagy is essential for adaptation to starvation in Drosophila
Source: Life Sci Alliance. 2020 Dec 14;4(2):e202000965. doi: 10.26508/lsa.202000965 (PMC7756965; doi:10.26508/lsa.202000965)
Supplement: Supplementary file 2 [file LSA-2020-00965_TableS2.docx]

**Table 2.** Genotype of the flies used in the experiments.

| **Figure 1** | |
| --- | --- |
| Fig 1B | *Cg-GAL4 UAS-Aru-GFP/LAMP1-3x mCherry* |
| Fig 1C, D | *w^1118^* |
| Fig 1E, F | *w^1118^*  *w^1118^;spin^EP822^*  *w^1118^;spin^P1^*  *w^1118^;cathD^1^*  *w^1118^;cathD^24/+^* |
| Fig 1G-I | *w^1118^*  *w^1118^;hsc70-4^∆19^/ Df(3R)BSC471* |
| Fig 1J | *w^1118^;hsc70-4^∆19^/ Df(3R)BSC471*  *w^1118^;hsc70-4^∆19^/ Df(3R)BSC471 hsc70-4^+^* |
| Fig 1L-O | *Cg-GAL4/UAS-Flag-Aru^WT^*  *Cg-GAL4/UAS-Flag-Aru^AA^* |
| **Figure 2** | |
| Fig 2A, C | *w^1118^* |
| Fig 2B | *Cg-GAL4 UAS- Aru-GFP* |
| Fig 2D-F | *w^1118^*  *w^1118^;aru^8-128^*  *w^1118^;aru^d8896^* |
| Fig 2G | *w^1118^;aru^8-128^*  *w^1118^;aru^8-128^;UAS-Aru-GFP* |
| Fig 2H, I | *w^1118^*  *w^1118^;aru^8-128^*  *w^1118^;aru^8-128^;UAS-Aru-GFP* |
| **Figure 3** | |
| Fig 3A, B | *Cg-GAL4 UAS-Aru-GFP* |
| Fig 3C, D | *w^1118^* |
| Fig 3E, F | *w^1118^*  *w^1118^;tor^∆P/+^* |
| Fig 3G, H | *HS-GAL4/+*  *HS-GAL4/+;UAS-TOR^TED^/+* |
| Fig 3I-L | *w^1118^*  *w^1118^;aru^8-128^*  *w^1118^;aru^d8896^* |
| **Figures 4 and 6** | |
| All panels | *w^1118^*  *w^1118^;aru^8-128^*  *w^1118^;aru^d8896^* |
| **Figure 5** | |
| Fig 5A-G | *w^1118^*  *w^1118^;aru^8-128^*  *w^1118^;aru^d8896^* |
| Fig 5H-K | *w^1118^*  *w^1118^;aru^8-128^*  *w^1118^;aru^8-128^;UAS-Aru-GFP* |
| **Figure S2** | |
| Fig S2A-C | *Cg-GAL4 UAS-Aru-GFP/LAMP1-3x mCherry* |
| Fig S2D |  |
| Fig S2E-G | *HS-GAL4/+;UAS-GFPnls/+*  *HS-GAL4/+;UAS-Atg1-RNAi/+*  *HS-GAL4/+;UAS-Atg13-RNAi/+* |
| Fig S2I | *Cg-GAL4/+*  *Cg-GAL4/+;UAS-HA-Hsc70-4^WT^/+*  *Cg-GAL4/+;UAS-HA-Hsc70-4^3KA^/+* |
| Fig S2J-M |  |
| **Figure S3** | |
| Fig S3C | *w^1118^* |
| Fig S3E | *w^1118^*  *Atg8a* |
| Fig S3F | *w^1118^*  *Atg7^∆77/∆14^* |
| **Figure S4** | |
| Fig S4A | *yw hs-flp;UAS-mCherry-Atg8a/+;Ac>mCD2>GAL4/UAS-luc-RNAi* |
| Fig S4B | *yw hs-flp;UAS-mCherry-Atg8a/+;Ac>mCD2>GAL4/UAS-aru-RNAi* |
| Fig S4C | *yw hs-flp;UAS-mCherry-GFP-Atg8a/+;Ac>mCD2>GAL4/UAS-luc-RNAi* |
| Fig S4D | *yw hs-flp;UAS-mCherry-GFP-Atg8a/+;Ac>mCD2>GAL4/UAS-aru-RNAi* |
| Fig S4E-J | *w^1118^*  *w^1118^;aru^8-128^*  *w^1118^;aru^d8896^* |
| **Figure S6** | |
| All panels | *w^1118^*  *w^1118^;aru^8-128^*  *w^1118^;aru^d8896^* |
| **Figure S7** | |
| All panels | *w^1118^*  *w^1118^;hsc70-4^∆19^/ Df(3R)BSC471*  *w^1118^;hsc70-4^∆19^/ Df(3R)BSC471 hsc70-4^+^* |
