# Supplementary material for: Degradation of arouser by endosomal microautophagy is essential for adaptation to starvation in Drosophila
Source: Life Sci Alliance. 2020 Dec 14;4(2):e202000965. doi: 10.26508/lsa.202000965 (PMC7756965; doi:10.26508/lsa.202000965)
Supplement: Supplementary file 3 [file LSA-2020-00965_TableS3.docx]

**Table 3.** Sequences for the primers used for RT-qPCR

| **gene** | **Forward primer sequence** | **Reverse primer sequence** |
| --- | --- | --- |
| *aru (CDS)* | CTGGAGAAGACCACGGGTATT | GCGGGGAATCTTTCGATTATGT |
| *aru-RD* | ACGTTTATTTTCCGCAAGCGA | GCTCCGTCTGTCTTCGCC |
| *aru-RA* | AGTTCAACTCGACCTAAGAAGC | CGTCTGAAGCTCCGTCGTTT |
| *hsc70-4* | CCAGGGTAATCGTACCACTCC | GCGTCGAAGATCGTCTGGG |
| *AkhR* | CCGTGCTGTATCTGCTGACC | CAGCGTCACCATTAGATCGG |
| *mcad* | TGGCACCTCTTTCGCACTC | GATGATCTCCTCACGGGTGAA |
| *bmm* | GTCTCCTCTGCGATTTGCCAT | CTGAAGGGACCCAGGGAGTA |
| *SREBP* | AGTCGCCGCTTCTCGTCTA | TGTATGGTGGCTGTTGGTTGG |
| *Acc* | ACCACCGAGGAGTTCGTGAA | CCGCTCGTTCTTAAACATTTCGT |
| *Fas* | GACATGGTCAACGATGATCCC | ACCGAAGAACTGTTGGTCAAAG |
| *lipin* | CACACCGACAACACACTGGA | CTTCTTCTCGCCCTGAAACAG |
| *CdsA* | ACAAAACGCCCGAGATATTGG | CGAAGCCGCAGATCATAATCC |
| *dilp6* | CCCTTGGCGATGTATTTCCCA | CTTGCAGCACAAATCGGTTAC |
| *dilp2* | CGAGGTGCTGAGTATGGTGTG | CCCCAAGATAGCTCCCAGGA |
| *Rp49* | AAGAAGTTCCTGGTGCACAACGTG | AATCTCCTTGCGCTTCTTGGAGGA |
