# Supplementary material for: Degradation of arouser by endosomal microautophagy is essential for adaptation to starvation in Drosophila
Source: Life Sci Alliance. 2020 Dec 14;4(2):e202000965. doi: 10.26508/lsa.202000965 (PMC7756965; doi:10.26508/lsa.202000965)
Supplement: Supplementary file 4 [file LSA-2020-00965_TableS4.docx]

**Table 4.** Descriptive P-values for quantification

| Figure 1D  Figure 1F  Figure 1H  Figure 1K  Figure 1N  Figure 1O | 0 vs. 2.5: * P=0.0133  "0 vs. 5: *** P=0.0009  *w^1118^* vs. *spin^EP^*: * P=0.0492  *w^1118^* vs. *spin^P1^*: * P=0.0215  *w^1118^* vs. *cathD^1^*: * P=0.0282  *w^1118^* vs. *cathD^24^*: ns P=0.3398  ** P=0.0047  *** P=0.0008  *w^1118^* vs. Flag-AruWT: *** P=0.0005  *w^1118^* vs. Flag-AruAA: *** P=0.0006  Flag-AruWT vs. Flag-AruAA: ns P=0.4191  *w^1118^* vs. Flag-AruWT: ns P=0.7032  *w^1118^* vs. Flag-AruAA: ns P=0.5279  Flag-AruWT vs. Flag-AruAA: ns P=0.9346 |
| --- | --- |
| Figure 2B  Figure 2D  Figure 2E  Figure 2F  Figure 2G  Figure 2I | ns: P=0.4125  **** P <0.0001  **** P <0.0001  *w^1118^* vs. *aru^8-128^*: * P=0.0404  *w^1118^* vs. *aru^8896^*: * P=0.0363  **** P <0.0001  **** P <0.0001 |
| Figure 3B  Figure 3D  Figure 3F  Figure 3H  Figure 3J  Figure 3K | Vehicle vs Rapamycin: ** P=0.0036  Vehicle vs Torin-1: * P=0.0375  Vehicle vs Rapamycin: ** P=0.0011  Vehicle vs Torin-1: * P=0.0121  *w^1118^* vs *tor^∆P/+^*: P=0.07  no HS vs +HS: * P=0.0292  *w^1118^*, fed vs starved: ** P=0.00309  *w^1118^*, fed vs starved: ** P=0.00542  *aru^8-128^*, fed vs starved: ** P=0.003646  *aru^8896^*, fed vs starved: ** P=0.001899 |
| Figure 4A  Figure 4B | *w^1118^*, fed vs starved: * P=0.017363  *aru^8-128^*, fed vs starved: ns P=0.410311  *aru^8896^*, fed vs starved: ns P=0.096569  *w^1118^*, fed vs starved: * P=0.016154  *aru^8-128^*, fed vs starved: ns P=0.183989  *aru^8896^*, fed vs starved: ns P=0.713503 |
| Figure 5B  Figure 5D  Figure 5E  Figure 5I  Figure 5J | *w^1118^* vs. *aru^8-128^*: **** P<0.0001  *w^1118^* vs. *aru^8896^*: **** P<0.0001  *w^1118^* vs. *aru^8-128^*: *** P=0.0001  *w^1118^* vs. *aru^8896^*: ** P=0.0094  *w^1118^* vs. *aru^8-128^*: * P=0.0167  *w^1118^* vs. *aru^8896^*: * P=0.0237  *w^1118^* vs. *aru^8-128^*: **** P<0.0001  *w^1118^* vs. rescue: * P=0.0228  *aru^8-128^* vs. rescue: *** P=0.0006  *w^1118^* vs. *aru^8-128^*: ** P=0.0094  *w^1118^* vs. rescue: ns P=0.4416  *aru^8-128^* vs. rescue: * P=0.0411 |
| Figure 6B  Figure 6C  Figure 6D  Figure 6E | *w^1118^*, ND vs. *aru^8-128^*, ND: ns P= 0.0819  *w^1118^*, ND vs. *aru^8896^*, ND: * P=0.0485  *w^1118^*, HSD vs. *aru^8-128^*, HSD: **** P <0.0001  *w^1118^* HSD vs. *aru^8896^*, HSD: *** P=0.0002  *w^1118^*, ND vs. *w^1118^*, HSD: * P=0.0136  *aru^8-128^*, ND vs. *aru^8-128^*, HSD: ns P=0.3950  *aru^8896^*, ND vs. *aru^8896^*, HSD: ns P=0.4603  Control vs *aru^8-128^*: *** P=0.0005  Control vs *aru^8896^*: **** P<0.0001  *aru^8-128^* vs *aru^8896^*: ns P=6380  Control vs *aru^8896^*: **** P<0.0001  Control vs *aru^8896/+^*: ns P=0.8295  *aru^8896/+^* vs *aru^8896^*: **** P<0.0001  elav-G4/+ vs elav-G4/UAS-Aru-RNAi: ns P>0.9999  elav-G4/+ vs UAS-Aru-RNAi/+: ns P=0.5095  elav-G4/UAS-Aru-RNAi vs UAS-Aru-RNAi/+: ns P>0.9999  ADH-G4/+ vs ADH-G4/UAS-Aru-RNAi: **** P<0.0001  ADH-G4/+ vs UAS-Aru-RNAi/+: ns P=0.5095  ADH-G4/UAS-Aru-RNAi vs UAS-Aru-RNAi/+: **** P<0.0001 |
| Figure EV2B  Figure EV2C  Figure EV2E  Figure EV2M | * P=0.0459  **** P<0.0001  ctl vs. Atg1-IR: * P=0.0366  ctl vs. Atg13-IR: * P=0.0115  ctl vs. *aru^8-128^*: ns P=0.2205  ctl vs. *aru^8896^*: ns P=0.8586 |
| Figure EV5 | *w^1118^*, ND vs. *aru^8-128^*, ND: **** P<0.0001  *w^1118^*, ND vs. *aru^8896^*, ND: **** P<0.0001  *w^1118^*, ND vs. *w^1118^*, HSD: * P=0.0172  *w^1118^*, ND vs. *aru^8-128^*, HSD: **** P<0.0001  *w^1118^*, ND vs. *aru^8896^*, HSD: **** P<0.0001 |
| Figure S2B | *w^1118^* vs. *hsc70-4*: ns P=0.6736  *w^1118^*vs. rescue: ns P=0.5394 |
